# Solid lipid nanoparticles as carriers for *Ulva rigida* peptide extract with demonstrated anti-β-amyloid and antioxidant properties

**DOI:** 10.3389/fnagi.2026.1778124

**Published:** 2026-03-13

**Authors:** Tânia G. Tavares, Iva Carvalho, Maria João Ramalho, Stéphanie Andrade, Maria Carmo Pereira, Joana A. Loureiro

**Affiliations:** 1 LEPABE—Laboratory for Process Engineering, Environment, Biotechnology and Energy, Faculty of Engineering, University of Porto, Porto, Portugal; 2 ALiCE—Associate Laboratory in Chemical Engineering, Faculty of Engineering, University of Porto, Porto, Portugal; 3 Department of Mechanical Engineering, Faculty of Engineering, University of Porto, Porto, Portugal

**Keywords:** Alzheimer’s disease, drug delivery, macroalgae peptides, nanoencapsulation, peptides extraction

## Abstract

**Introduction:**

Alzheimer’s disease (AD) remains one of the greatest challenges in neurodegenerative research, largely due to the toxic aggregation of the β-amyloid (Aβ) peptide and the difficulty of delivering therapeutic compounds across the blood-brain barrier (BBB).

**Methods:**

This work explored *Ulva rigida* (*U. rigida*), a green macroalga rich in bioactive peptides, as a natural source of anti-amyloidogenic and antioxidant agents.

**Results:**

First, the peptides extraction process was optimized by design of experiments (DoE). Peptides extracted and hydrolyzed under optimized conditions showed antioxidant capacity (2.51 ± 0.18 μmol_Trolox equivalent_/ mg_hydrolyzed protein_), and the fraction of peptides with a molecular weight lower than 3 kDa was able to reduce the aggregation of Aβ_*1–42*_ peptide *in vitro* by around 60%. To overcome the issue of limited bioavailability, we encapsulated these peptides in solid lipid nanoparticles (SLNs). The developed nanoformulation displayed a size of 158 ± 14 nm, narrow polydispersity, and near-neutral zeta potential (−4.7 ± 0.6 mV), with an encapsulation efficiency (EE) of 54.1 ± 0.1% and suitable stability under simulated gastrointestinal and storage conditions.

**Discussion:**

By combining biological activity with a delivery platform, this study highlights the potential of *Ulva rigida* as a source of bioactive peptides with the potential to delay the progression of AD.

## 1 Introduction

Alzheimer’s disease (AD) is a brain disorder that is the main cause of dementia around the world. Currently, it is estimated to affect approximately 55 million people, with about 75% experiencing cognitive difficulties (Alzheimers Dementia, 2024). Clinically, this manifests as progressive cognitive decline, including memory impairment, confusion, and deficits in executive function. As people live longer, AD is becoming a significant health problem in this century. Although scientists have been studying it for years, no treatments can significantly alter the course of the disease (The Lancet Neurology, 2024). Current pharmacological interventions for AD are limited to symptomatic relief, primarily targeting memory loss, without addressing the underlying neuropathological mechanisms. AD has two hallmark features: extracellular deposits of β-amyloid (Aβ) peptides forming senile plaques, and intracellular neurofibrillary tangles composed of hyperphosphorylated tau protein. These pathological changes disrupt neuronal communication and function, ultimately leading to cell death. Accumulating evidence suggests that Aβ aggregation is among the earliest events in the pathogenesis of AD, underscoring the urgent need for disease-modifying therapies (Dubois et al., 2023; Selkoe and Hardy, 2016). Aβ peptide is derived from the cleavage of amyloid precursor protein (APP), initially existing as monomeric units in the extracellular space. These monomers aggregate into oligomers and larger fibrillar structures, which are neurotoxic and contribute to neuronal dysfunction. The accumulation of Aβ aggregates induces oxidative stress, disrupts calcium homeostasis, and promotes neuroinflammation, ultimately leading to neuronal damage (O’Brien and Wong, 2011). Current pharmacological approaches have limited efficacy in preventing Aβ aggregation (Zhang et al., 2023), prompting researchers to investigate alternative therapeutic strategies, including the use of bioactive natural compounds.

Marine macroalgae are gaining increased attention, since these seaweeds can provide different molecules, such as polysaccharides, polyphenols, and proteins. Among these, bioactive peptides (BAPs) could be useful because they can fight stress and swelling, protect brain cells, lower blood pressure, and work as antimicrobial agents (Monroy-García et al., 2025; Negreanu-Pirjol et al., 2022). Small peptides have garnered significant interest due to their ability to modulate physiological processes such as neuroinflammation and oxidative stress. However, their clinical application faces notable challenges. Peptides are prone to rapid enzymatic degradation *in vivo*, which limits their stability and bioavailability (Fosgerau and Hoffmann, 2015).

The application of nanotechnology in biomedical research has opened new opportunities for addressing drug delivery challenges, particularly in treating neurological disorders. Among the most promising tools are nanoparticles (NPs), which are versatile carriers capable of enhancing drug stability and facilitating targeted delivery. Due to their physicochemical properties, NPs can traverse biological barriers, including the BBB, allowing therapeutic agents to reach the CNS more effectively (Andrade et al., 2022a; Ramalho et al., 2023).

Lipid-based drug delivery systems have been widely investigated as effective strategies to enhance the bioavailability, stability, and brain delivery of phytocompounds with neuroprotective potential (Dirir et al., 2025; Fernandes et al., 2021; Ozay and Karpuz, 2024). In particular, solid lipid nanoparticles (SLNs) have gained attention as drug delivery systems for their biocompatibility, structural integrity, and ability to encapsulate a wide range of compounds. These lipid-based systems comprise a solid core surrounded by stabilizing agents, enabling controlled and sustained drug release, making them especially suitable for complex therapeutic formulations. Furthermore, their small size and cellular permeability contribute to prolonged circulation times and reduced clearance, enhancing their potential in neuropharmacological applications (Gonçalves et al., 2021; Pereira et al., 2022; Satapathy et al., 2021). These characteristics position SLNs and other nanocarriers as promising candidates for overcoming current limitations in brain-targeted therapies, offering a platform for more efficient and precise treatment strategies.

Several marine-derived and natural bioactive peptides with anti-amyloidogenic activity have been reported in recent years, highlighting the therapeutic potential of marine resources for AD (Kabir et al., 2021). In parallel, different nanocarrier-based delivery systems have been explored to improve peptide stability and bioavailability (Mehrdadi, 2024). However, most studies have focused either on isolated peptides without delivery optimization or on nanoencapsulation strategies lacking systematic peptide enrichment, molecular weight control, or combined evaluation of antioxidant and anti-Aβ aggregation activities. In particular, the integration of low-molecular-weight marine peptide fractions with SLN remains underexplored. In this study, bioactive peptides were extracted from the green macroalga *Ulva rigida* (*U. rigida*) by applying an experimental design to optimize the content of peptides with weights lower than 3 kDa. The *in vitro* activity of the peptide extract concentrate (lower than 3 kDa) was evaluated, with particular focus on its antioxidant properties and its ability to inhibit the aggregation of the Aβ_1–42_ peptide. Due to the inherent instability of peptides and their limited permeability across the BBB, the BAP with low molecular weight was subsequently encapsulated into SLNs. The developed nanocarriers were characterized in terms of particle size, polydispersity index, zeta potential, encapsulation efficiency (EE), loading capacity (LC), and physicochemical stability under simulated gastrointestinal and storage conditions.

Unlike previous studies that primarily focus on isolated marine peptides or single-function nanoformulations, the present work combines an optimized low-molecular-weight peptide fraction (<3 kDa) from *U. rigida* with a lipid-based nanocarrier system. This integrated strategy enables the simultaneous assessment of antioxidant and anti-amyloidogenic activities, while addressing peptide instability and delivery limitations through solid lipid nanoparticle encapsulation.

The integration of marine-derived bioactive peptides with nanotechnology represents a promising and sustainable approach to address the multifactorial challenges of AD treatment.

## 2 Materials and methods

### 2.1 Materials

*Ulva rigida* was acquired from ALGAplus, Portugal. Ethanol (EtOH, Mw 46.07, 99.9%), sodium hydroxide (NaOH, Mw 40.00, 98.9%), hydrochloric acid (HCl, Mw 36.46, 35.9%), sodium sulfite (Na_2_SO_3_, Mw 126.04, 97.8%), potassium hydrogen phosphate (K_2_HPO_4,_ Mw 174.18, 99.3%), sodium phosphate monobasic monohydrate (NaH_2_PO_4_⋅H_2_O, Mw 137.99, ≥ 99.0%), methanol (MeOH, Mw 32.04), sodium chloride (NaCl, Mw 58.44, ≥ 98.0%), fluorescein sodium salt (FL) (Mw 376.28, ≥ 95.0%), Tris (hydroxymethyl) aminomethane hydrochloride (Tris-HCl, Mw 157.60, ≥ 99.0%), phosphate buffered saline (PBS, pH 7.4, KCl 2.7 mM, NaCl 140 mM, phosphate 10 mM), and zinc chloride (ZnCl_2_, Mw 136.28) were purchased from VWR Chemicals, Germany. Pluronic F-127 (Mw 12,600), flavourzyme (Protease form Aspergillus oryzae, Mw 27.29), trinitrobenzenesulfonic acid (5% (w/v) in H_2_O (TNBS) (Mw 293.17), bovine serum albumin (BSA, Mw ∼66,000), and thioflavin T (ThT, Mw 318.86), were purchased from Sigma Aldrich, St. Louis, MO, USA. L-leucine (Mw 131.18) was supplied by Merck KGaA (China), 2,2’-azobis-2-methyl-propanimidamide dihydrochloride (AAPH, Mw 271,19) from Cayman Chemical Company (Michigan, United States), ( ± )-6-hydroxy-2,5,7,8-tetramethylchroman-2-carboxylic acid (Trolox, Mw 250.29) from EMD Chemicals (San Diego, United States), o-aminobenzoylglycyl-p-nitrophenylalanylproline (Mw 483.47) from Bachem Feinchemikalien (Bubendorf, Switzerland), uranyl acetate (Mw 424.14, 99.6%) from Electron Microscopy Science (Hatfield, PA, United States), and FaSSGF Buffer Concentrate, FaSSIF Buffer Concentrate and 3F Powder were acquired from Biorelevant (United Kingdom). The Pierce*™* BCA protein assay kit was bought from Thermo Scientific (Rockford, United States). The lipids Precirol^®^ 5 ATO, Compritol^®^ 888 ATO, Apifil^®^, Gelucire^®^ 50/13, Softisan^®^ 100, and Compritol^®^ HD5 ATO were provided by the Gatefossé SAS, France. Human amyloid-β peptide (1–42) (Aβ_1–42_, purity > 95%, MW 4514.08) was from GenScript Biotech (Piscataway, NJ, United States).

### 2.2 Seaweed protein extraction

The seaweed used in this study was *U. rigida*. According to the supplier’s specification sheet, the macronutrients present in the macroalgae sample are 19.6 g of protein, 1.1 g of lipids, 49.1 g of carbohydrates, and 5.4 g of salt.

#### 2.2.1 Seaweed cell disruption

A pre-treatment was performed by mixing 250 mg of freeze-dried *U. rigida* with 10 mL of pure EtOH. The mixture was gently stirred to ensure complete contact between the biomass and the solvent, and subsequently centrifuged at 8,000 rpm for 10 min at 5 °C (Model 1580R, Gyrozen, Gimpo, South Korea). The resulting pellet was subjected to cell disruption using a homogenizer (Precellys Evolution System, Bertin Instruments, Montigny-le-Bretonneux, France). Each vial contained the EtOH-treated pellet, eight ceramic beads, and 5 mL of ultrapure water. Homogenization was carried out in six cycles of 30 s at 8,000 rpm with 45 s intervals between cycles. An additional 5 mL of ultrapure water was then added, and the same homogenization program was repeated. After disruption, the samples were centrifuged at 8,000 rpm for 10 min at 5 °C (1580R, Gyrozen), and the supernatants were collected and freeze-dried under vacuum until complete dehydration using a bench-top freeze-dryer (Model 6K, VirTis, New York, NY, United States).

#### 2.2.2 Optimization of conventional seaweed protein extraction

Protein extraction from *U. rigida* was optimized using response surface methodology (RSM) with a central composite design (CCD) using the Design Expert software (Version 13, Stat-Ease, Minneapolis, MN, United States). The effects of NaOH concentration, incubation time (IT), and temperature (T) were evaluated on protein yield. The CCD comprised 20 experimental runs, including 8 factorial points (−1 and +1), 6 axial points ( ± 1.68), and 6 central points for variance estimation. The tested ranges were 0.1–0.4 M NaOH, 15–50 min, and 15–40 °C for NaOH concentration, IT, and T, respectively. Analysis of variance (ANOVA) was applied, and model terms were considered significant at *p* < 0.05. Model fitting was evaluated based on the coefficients of determination (R^2^, adjusted R^2^, predicted R^2^). Optimal conditions were determined by maximizing the desired response variable.

Pellets obtained from the pre-treatment were randomly subjected to the CCD conditions (Table 1). After extraction, samples were centrifuged at 8,000 rpm for 10 min at 5 °C (1580R, Gyrozen). Protein concentration in the supernatants was determined using a BCA assay kit with BSA as the standard. Measurements were performed in 96-well plates, and absorbance was read at 562 nm using a microplate reader after 30 min of incubation at 37 °C (FLUOstar^®^ Omega, BMG Labtech, Ortenberg, Germany). Once the optimal extraction conditions were established, the process was repeated under these parameters. The resulting supernatant was purified and concentrated by diafiltration using a Macrosep^®^ Advance device (1 kDa MWCO; Pall Life Sciences, Cork, Ireland) at 8,000 rpm and 5 °C (1580R, Gyrozen).

**TABLE 1:** The experimental design covering the experimental independent variables NaOH concentration, incubation time (IT), and temperature (T)—and the results refer to a response variable—% protein obtained for Ulva rigida extract.

Experiment	NAOH (M)	IT (min)	T (°C)	Protein (%)
1	0.40	50.0	25.0	7.79 ± 0.37
2	0.25	32.5	32.5	7.63 ± 0.41
3	0.25	32.5	45.1	8.95 ± 0.02
4	0.25	32.5	32.5	8.20 ± 0.07
5	0.50	32.5	32.5	8.61 ± 0.25
6	0.00	32.5	32.5	2.53 ± 0.04
7	0.10	50.0	40.0	8.38 ± 0.44
8	0.40	15.0	40.0	7.95 ± 0.09
9	0.25	61.9	32.5	7.88 ± 0.02
10	0.40	15.0	25.0	7.35 ± 0.01
11	0.25	32.5	32.5	7.70 ± 0.21
12	0.25	32.5	32.5	7.91 ± 0.08
13	0.25	3.1	32.5	5.59 ± 0.25
14	0.25	32.5	32.5	7.54 ± 0.16
15	0.25	32.5	32.5	7.80 ± 0.07
16	0.25	32.5	19.9	6.95 ± 0.01
17	0.10	50.0	25.0	6.66 ± 0.15
18	0.10	15.0	40.0	6.39 ± 0.10
19	0.40	50.0	40.0	9.72 ± 0.08
20	0.10	15.0	25.0	6.09 ± 0.05

### 2.3 Seaweed protein enzymatic hydrolysis

The enzymatic hydrolysis of *U. rigida* protein extracts was also optimized using a RSM, as described in section 2.2.2. The evaluated parameters were the enzyme-to-substrate (E/S) ratio (v/v) and reaction time (RT), while the response variables were the degree of hydrolysis (DH) and *in vitro* antioxidant capacity. The CCD comprised 13 experiments, including 4 factorial points (−1 and +1), 4 axial points ( ± √2), and 5 central points for variance estimation. The tested ranges were 0.1–2.9 (E/S ratio) and 0–7 h (RT).

Substrate solutions were prepared by mixing the filtrate from the NaOH extraction with the freeze-dried powder obtained from the Precellys water extraction. The pH was adjusted to 7.5 using 5 M HCl, and hydrolysis was performed with flavourzyme at 50 °C for up to 7 h. Samples were withdrawn at 0, 1, 3.5, 6, and 7 h, and the reaction was stopped by heating at 95 °C for 10 min. The hydrolysates were centrifuged at 5,000 rpm for 15 min at 4 °C, and the peptide extract concentrate was obtained and subjected to fractionation using a Macrosep^®^ Advance device (3 kDa MWCO; Pall Life Sciences) at 8,000 rpm and 5 °C (1580R, Gyrozen). All obtained fractions were stored at −20 °C.

The DH of the TPH was determined by measuring free amino groups using the TNBS method (Gonçalves et al., 2021). Briefly, 0.05 mL of the sample was mixed with 0.2 mL of 0.015% (w/v) TNBS and 0.5 mL of 1 M potassium borate buffer (pH 9.2) and incubated for 30 min at 25 °C in the dark. The reaction was stopped with 0.2 mL of 2 M NaH_2_PO_4_ containing 18 mM Na_2_SO_3_, and absorbance was measured at 420 nm using a spectrophotometer (UV-1800, Shimadzu, Kyoto, Japan). L-leucine was used to construct the standard curve (0–2 mM), and DH (%) was calculated using Equation 1 (Baek and Cadwallader, 1995):


$$DH\left(\%\right)=\frac{L_{i}-L_{0}}{L_{m\,a\,x}}-L_{0}\times 100\%$$ (1)

where *L*_*i*_ is the amount of liberated amino acids in sample *i*, *L*_0_ is the number of amino acids in the original substrate (blank), and *L*_*max*_ is the maximum amount of the specific amino acids in the substrate obtained after hydrolysis.

### 2.4 Evaluation of the extract in vitro bioactivity

#### 2.4.1 Antioxidant capacity

The *in vitro* antioxidant capacity of the peptide extract concentrate was determined using the oxygen radical absorbance capacity (ORAC-FL) assay, which evaluates peroxyl radical scavenging capacity with fluorescein (FL) as a fluorescent probe (Satapathy et al., 2021). Phosphate buffer (0.075 M, pH 7.4) was used, and stock solutions of FL (1.17 mM) and AAPH (46.6 mM) were freshly prepared. Trolox (0–100 μM) was used to construct the calibration curve. Assays were performed in 96-well black plates (Thermo Fisher Scientific, Denmark) using a microplate reader (FLUOstar^®^ Omega, BMG LABTECH, Germany) with excitation and emission wavelengths of 485 and 520 nm, respectively. Each well contained 20 μL of sample or standard, 120 μL of FL solution, and 60 μL of AAPH solution. Blanks contained PBS instead of the sample. Reactions were carried out at 37 °C for 100 min in the dark, and fluorescence was recorded every minute.

The area under the fluorescence decay curve (AUC) was calculated using normalized fluorescence values, and the net AUC was obtained by subtracting the blank AUC. ORAC-FL values were expressed as μmol Trolox equivalents per mg of hydrolysed protein (μmol TE/mg protein) according to Kabir et al. (2021).

#### 2.4.2 Anti-amyloidogenic activity of the peptide extract concentrate

The *in vitro* anti-amyloidogenic activity of the peptide extract concentrate was monitored using a protocol previously established (Andrade et al., 2021). Briefly, aggregation kinetics were assessed by mixing the peptide extract concentrate with Aβ_1–42_ monomers at a concentration of 25 μM in PBS in a 96-well black plate (untreated, flat bottom, UV-Star^®^) using a molar ratio of Aβ_1–42_/extract of 1:25. Controls were also prepared, i.e., Aβ in the absence of the, PBS buffer or peptide extract concentrate. The samples were treated with a final concentration of 50 μM of ThT dissolved in PBS buffer. The microplate was covered to prevent evaporation and left to incubate for 10 h at 37 °C, under continuous medium stirring. Fluorescence readings were taken every minute using a Synergy™ 2 Multi-Mode microplate reader (Biotek, Germany) equipped with a 420/50 nm excitation filter and a 485/20 nm emission filter.

#### 2.4.3 Aβ fibril formation kinetic model

After collecting the ThT fluorescence readings, the signal from the corresponding blanks was subtracted from each sample. The normalization of the data was performed based on the control data (Aβ). The resulting curves were then fitted to a sigmoidal model (Equation 2), in which the normalized fluorescence intensity *F(t)* is expressed as a function of time *t* (hours):


$$F\,\left(t\right)=F_{0}+\frac{F_{m\,a\,x}}{1+e^{\left[-k\,\left(t-t_{\frac{1}{2}}\right)\right]}}$$ (2)

*F0* denotes the initial fluorescence intensity at the onset of the assay, while *F*_*max*_ corresponds to the maximum fluorescence reached during aggregation. The parameters *k* and *t_1/2_* describe the kinetic profile of fibril formation, representing the elongation rate constant and the time required to reach half of the maximum fluorescence, respectively.

From these fitted parameters, the lag phase of amyloid fibril growth (*t*_*lag*_) was calculated using Equation 3.


$$t_{l\,a\,g}=t_{1/2}-\frac{2}{k}$$ (3)

### 2.5 Preparation of solid lipid nanoparticles

#### 2.5.1 Lipid screening

A suitable solid lipid was chosen to serve as the matrix for preparing the SLN dispersion. For this purpose, 0.5 mg of the peptide extract with a molecular weight lower than 3 kDa was mixed with 25 mg of solid lipid. The mixtures were placed in a bath at 80 °C (temperature above the melting point of the solid lipid), and the compatibility of the extract with different solid lipids was evaluated at intervals of 15–60 min. The solid lipids tested were Precirol^®^ 5 ATO, Gelucire^®^ 50/13, Compritol^®^ 888 ATO, Softisan^®^ 100, Apifil^®^ and Compritol^®^ HD5 ATO. The compatibility was evaluated by a visual test, analyzing whether the extract is solubilized in the lipid phase.

#### 2.5.2 Preparation of peptide extract concentrate-loaded SLNs

SLNs were prepared using a high-shear homogenization process followed by ultrasonication. The experimental procedure was adapted from the method described by Gonçalves et al. (2021). Initially, 250 mg of the solid lipid was mixed with 100 mg of the extract and heated to 80 °C. Separately, 2.175 mL of an aqueous solution of Pluronic F-127 (10% w/v) was heated to the same temperature. Once the lipid was fully melted, the aqueous phase was added to the lipid phase containing the extract. The obtained mixture was then homogenized for 1 min using an Ultra-Turrax T25 homogenizer (Janke and Kunkel IKA-Labortechnik, Staufen, Germany) operating at 13,500 rpm, followed by 5 min of sonication at 80% amplitude and a frequency of 24 kHz (ultrasonic processor UP400S, Hielscher, Germany). The resulting samples were cooled to room temperature under gentle magnetic stirring (100 rpm) and stored protected from light. Unloaded SLNs were also produced as control samples.

### 2.6 Characterization of the nanoparticles

The physicochemical characterization of the NPs was carried out by assessing their average diameter, polydispersity index (PdI), and zeta potential using Dynamic Light Scattering (DLS) combined with Laser Doppler Electrophoresis (ZetaSizer Nano ZS, Malvern Instruments, United Kingdom). Before analysis, the samples were diluted in ultrapure water at 1:100 (1.4 mg/mL). Measurements of particle size and PdI were performed in a polystyrene cuvette (Sarstedt^®^, United Kingdom), whereas zeta potential determinations employed a disposable folded capillary cell (DTS1070, Malvern Instruments, United Kingdom). Experimental parameters were established with water as the dispersant medium at 25 °C, using a refractive index of 1.330, viscosity of 0.8872 cP, and dielectric constant of 78.2. Data acquisition and processing were conducted using the ZetaSizer software (Malvern Instruments, United Kingdom).

SLNs-extract loaded morphology was examined by transmission electron microscopy (TEM). A 10 μL of SLNs diluted at 1:100 (1.4 mg/mL) was pipetted onto the brightest side of the grid and left to adsorb for 5 min, after which the excess sample was removed. The uranyl acetate solution was centrifuged for 3 min at 14,500 rpm (MiniSpin^®^plus, Eppendorf, Germany) before use. Finally, the grid was stained with 10 μL of uranyl acetate for 45 s, and after removing the excess of uranyl acetate, the grid was stored for later analysis. A JEM 1400 electron microscope (JEOL, Tokyo, Japan) operating at an accelerating voltage of 80 kV was used to obtain TEM micrographs.

### 2.7 Determination of the peptide extract concentrate encapsulation efficiency and NPs loading capacity

The EE and LC of the NPs were indirectly determined by quantifying the amount of unencapsulated peptide remaining in the supernatant. Briefly, 500 μL of extract-loaded NPs, previously diluted 1:20 (v/v), were passed through a PD MiniTrap desalting column containing Sephadex G-25 resin (Cytiva, United States). The absorbance values were correlated with a previously established calibration curve prepared in ultrapure water using a Synergy™ 2 Multi-Mode Microplate Reader (BioTek Instruments, Winooski, VT, United States). The encapsulation efficiency was then calculated using Equation 4.


$$EE\left(\%\right)=\frac{t\,o\,t\,a\,l\,m\,a\,s\,s\,o\,f\,a\,d\,d\,e\,d\,e\,x\,t\,r\,a\,c\,t-f\,r\,e\,e\,e\,x\,t\,r\,a\,c\,t\,m\,a\,s\,s}{t\,o\,t\,a\,l\,m\,a\,s\,s\,o\,f\,a\,d\,d\,e\,d\,e\,x\,t\,r\,a\,c\,t}\times 100$$ (4)

The LC, expressed as a percentage, represents the ratio between the mass of encapsulated peptide extract and the total mass of extract-loaded SLNs, as defined in Equation 5. The total mass of the SLNs was defined as the sum of the masses of Pluronic F-127, the solid lipid, and the encapsulated peptide extract.


LC(%)=

$$\frac{m\,a\,s\,s\,o\,f\,e\,n\,c\,a\,p\,s\,u\,l\,a\,t\,e\,d\,e\,x\,t\,r\,a\,c\,t}{t\,o\,t\,a\,l\,m\,a\,s\,s\,o\,f\,S\,L\,N\,s+t\,o\,t\,a\,l\,m\,a\,s\,s\,o\,f\,e\,n\,c\,a\,p\,s\,u\,l\,a\,t\,e\,d\,e\,x\,t\,r\,a\,c\,t}\times 100$$ (5)

### 2.8 Evaluation of nanoparticles’ stability

#### 2.8.1 Stability over the simulated gastrointestinal environment

Since oral administration was considered, the simulated gastric medium FaSSGF and FaSSIF buffers were used to evaluate the NPs’ behavior during the digestion (Minekus et al., 2014). For testing in each medium, the NPs suspensions were diluted 1:200 (v/v) in water. A 2 mL aliquot of the diluted sample was placed into a Spectra/Por Float-A-Lyzer G2 dialysis device, CE, 10 kDa (Spectrum Laboratories, Inc., Los Angeles, CA, United States) and immersed in 50 mL of FaSSGF buffer at 37 °C with continuous stirring at 200 rpm for 2 h. After incubation, a small portion of the sample was collected from inside the membrane to determine the NPs’ size, PdI, and zeta potential as described in 2.7. The analyzed sample was then returned to the dialysis device. After that, the membrane was re-immersed in 50 mL of FaSSIF buffer, under the same conditions (37 °C, 200 rpm) for 3 h. At the end of the incubation period, the NPs were again characterized in terms of size, PdI, and zeta potential.

#### 2.8.2 Stability over time under storage conditions

The stability of the prepared SLNs with the peptide extract encapsulated was investigated for 21 days at room temperature and at 4 °C. Changes in size, PDI, and zeta potential over time were used to evaluate their stability. The physicochemical properties of the NPs were evaluated as described in 2.6. The samples were also visually inspected to detect any presence of phase separation or flocculation, both of which are signs of stability issues.

### 2.9 Statistical analysis

Data are shown as the average ± standard deviation (SD) from a minimum of three independent tests. Student’s *t*-tests were conducted with a 95% confidence using Microsoft Excel.

## 3 Results and discussion

### 3.1 Protein extraction

**FIGURE 1:**
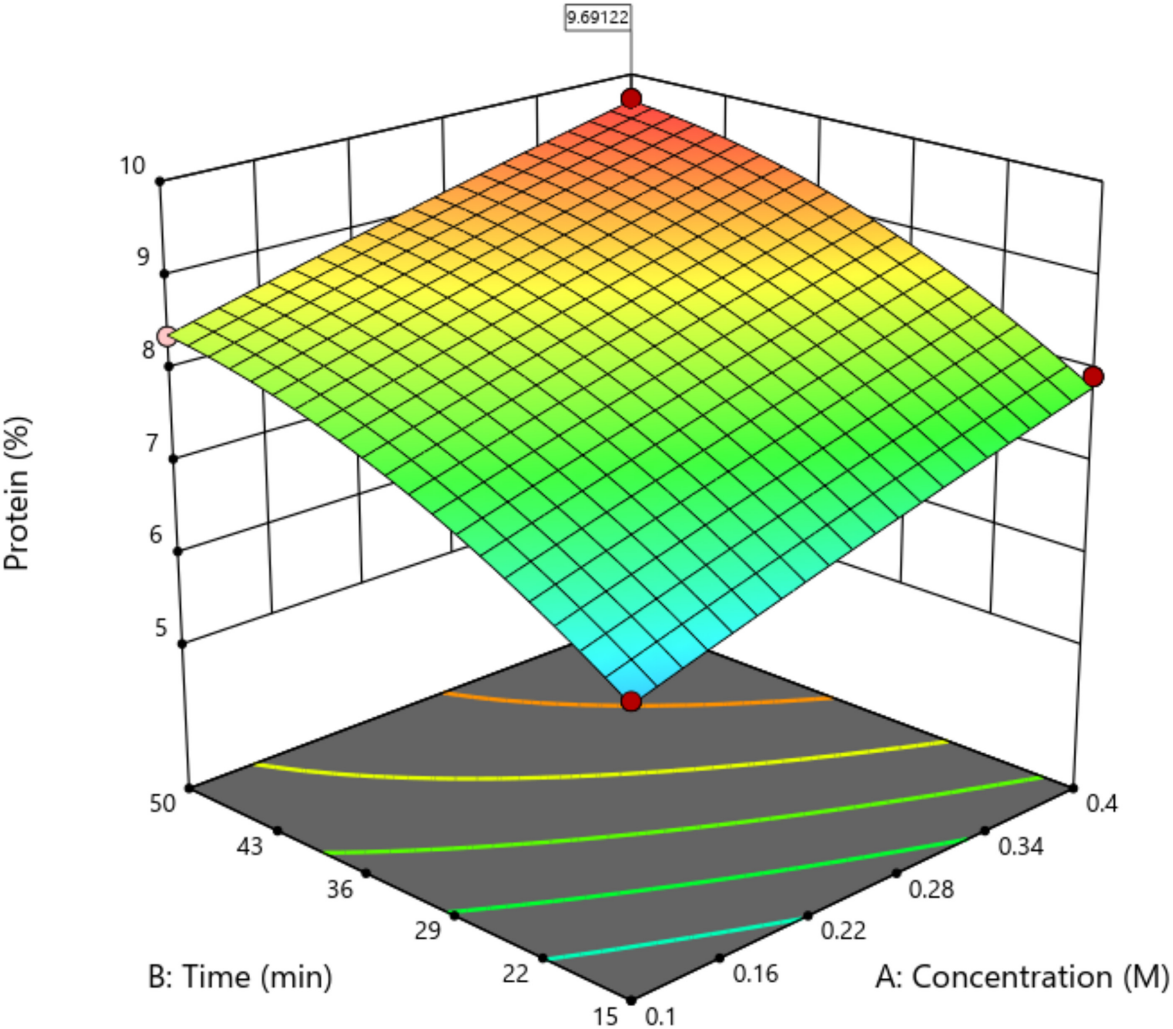
Variation of the predicted response for the percentage of protein depending on each term of the model, NaOH concentration, and IT, setting the temperature at 40 °C, obtained for the *U. rigida* extract.

Optimization of protein extraction from *U. rigida* was conducted using a CCD to evaluate the combined effects of NaOH concentration, IT, and T. The experiments, randomized across the variable ranges shown in Table 1, generated data that were fitted to a quadratic response surface model (Figure 1).

The regression analysis revealed a strong correlation between predicted and adjusted values, with R^2^ values of 0.9207 and 0.9565, respectively. Analysis of variance (ANOVA) confirmed the model’s statistical significance (*F* = 45.00, *p* < 0.0001), suggesting only a 0.01% likelihood that the model’s performance resulted from random variation. Significant model terms included NaOH concentration, IT, and T as primary variables, as well as IT × T (interaction) and IT^2^ (quadratic) terms (*p* < 0.05), demonstrating that both linear and interactive effects critically influenced protein extraction efficiency. The fitted quadratic model adequately described the experimental data within the studied range, indicating that protein extraction efficiency is governed by the combined influence of alkaline conditions and processing time–temperature parameters. The lack-of-fit test yielded a non-significant result (Prob > *F* = 0.7707), confirming that the model adequately represented the experimental data. Collectively, these findings validate the quadratic response surface model as a reliable predictive tool for optimizing conventional alkaline extraction of *U. rigida* proteins. Analysis of the response surface derived from the CCD (Figure 1) enabled prediction of protein extraction behavior as influenced by NaOH concentration and IT, with temperature maintained at 40 °C for comparative consistency. This condition was selected based on earlier reports demonstrating that elevated temperatures enhance protein solubilization by promoting cell wall loosening (Karabulut et al., 2024).

The response surface revealed that protein extraction increased with both NaOH concentration and IT, indicating that longer alkaline exposure facilitates matrix disruption of *U. rigida*. Similar trends were observed by Chandran et al. (2024) and Karabulut et al. (2024) who reported improved extraction yields at higher temperatures. However, prolonged incubation may lead to microbial proliferation, as well as risk protein aggregation if not carefully controlled, which can reduce both yield and functional properties (Görgüç et al., 2019; Karabulut et al., 2024).

Desirability analysis identified the optimal extraction conditions as 0.4 M NaOH, 40 °C, and 50 min. Validation under these conditions (six replicates) yielded a protein extraction of 9.6 ± 0.2%, closely matching the predicted 9.7 ± 0.2%, confirming the model’s robustness.

Considering the total protein content of *U. rigida* (19.6%), the extraction yield corresponded to roughly 50% of total protein, surpassing the values reported by Fleurence et al. for *U. rigida* and *Ulva rotundata* (26.8 ± 1.3% and 36.1 ± 1.4%, respectively) (Fleurence et al., 1995). Comparable results were obtained by Juul et al. for *Ulva fenestrata* using homogenization-assisted extraction (8.95 ± 0.79%) (Juul et al., 2021).

These findings reinforce that efficient protein recovery from *Ulva* relies on effective cell wall disruption. The optimized parameters proposed here represent a simple, rapid, and environmentally sound process for valorising *U. rigida* proteins for food and biotechnological applications.

### 3.2 Optimization of protein hydrolysis

Beyond temperature and pH, the most influential factors affecting enzymatic hydrolysis are the enzyme-to-substrate (E/S) ratio (v/v) and reaction time (RT, h). Therefore, these two parameters were systematically investigated by quantifying the degree of hydrolysis (DH) and the antioxidant capacity of the resulting seaweed protein hydrolysates, determined by the ORAC assay.

Experiments were carried out in randomized order across different combinations of E/S ratio and RT, within the ranges defined in Table 2, while also considering industrial feasibility. As summarized in Table 2, hydrolysates produced using flavourzyme exhibited the highest content of free amino groups, corresponding to a DH of 93.1 ± 0.8%. Regarding the antioxidant capacity, flavourzyme hydrolysates also displayed the highest ORAC value of 2.5 ± 0.2 μmol_Trolox equivalents_/mg_hydrolysed protein_.

**TABLE 2:** Experimental design covering two processing parameters, E/S ratio and reaction time (RT), results referring to two response variables, degree of hydrolysis (DH), and antioxidant capacity (ORAC).

Experiment	E/S ratio	RT (h)	DH (%)	Antioxidant capacity (μ mol_Trolox_ _equivalent_/mg_hydrolysed_ _protein_)
1	1.5	3.5	67.0 ± 6.2	2.02 ± 0.20
2	1.5	3.5	71.6 ± 5.5	1.98 ± 0.24
3	2.5	1.0	67.3 ± 6.3	2.09 ± 0.09
4	1.5	3.5	69.8 ± 3.0	1.94 ± 0.08
5	0.5	6.0	57.5 ± 0.3	1.92 ± 0.10
6	0.5	1.0	40.2 ± 2.5	1.74 ± 0.01
7	1.5	3.5	71.0 ± 2.9	2.21 ± 0.02
8	0.1	3.5	-5.4 ± 1.4	1.01 ± 0.01
9	2.5	6.0	93.1 ± 0.8	2.51 ± 0.18
10	2.9	3.5	92.5 ± 5.6	2.44 ± 0.22
11	1.5	0.0	0.2 ± 1.2	1.56 ± 0.07
12	1.5	7.0	81.5 ± 6.4	2.35 ± 0.16
13	1.5	3.5	63.4 ± 2.7	2.09 ± 0.16

**FIGURE 2:**
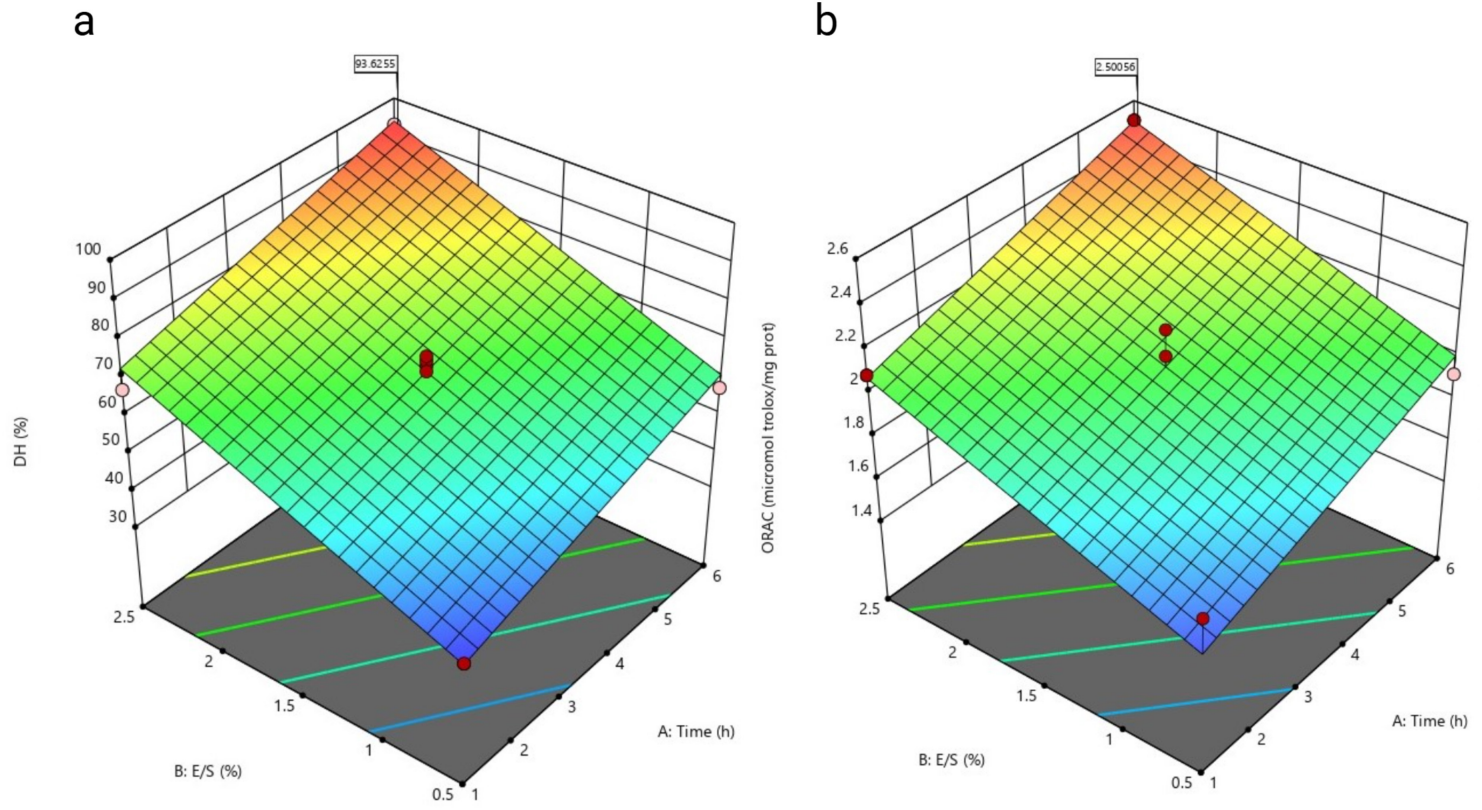
Variation of the predicted response to the **(a)** degree of hydrolysis (DH), and **(b)** antioxidant capacity, depending on each term of the model—time and E/S ratio, obtained for a hydrolysed extract of *U. rigida*.

Regression analysis was applied to fit the experimental data to a RSM, which was subsequently used to generate the response surface plots shown in Figure 2. These results confirm that both E/S ratio and reaction time strongly influence the efficiency of enzymatic hydrolysis and the bioactive potential of *U. rigida* protein hydrolysates.

Each response variable in the experimental design was fitted to the model that best described its behavior. The resulting regression analyses demonstrated strong predictive capacity, with coefficients of determination (R^2^) of 0.9657 for DH and 0.8665 for antioxidant capacity. These results indicate that the linear response surface models provided an adequate fit to the experimental data.

The adequacy and statistical significance of the models were further evaluated through analysis of variance (ANOVA) employing Fisher’s F-test. The F-values obtained were 112.59 for DH and 29.21 for antioxidant capacity, both exceeding the critical limits for significance (*p* < 0.001). The probability that such high F-values could result from random variation was only 0.01%, confirming the robustness of the models. In both models, experiment 8 was excluded from the statistical analysis due to its markedly low response values, which deviated from the model’s predictive range. Nevertheless, these data remain biologically relevant, as they indicate that enzymatic hydrolysis did not occur under those specific experimental conditions. This observation provides valuable confirmation of the critical thresholds required for effective enzyme activity and supports the model’s overall interpretive consistency. In both models, the independent variables, E/S ratio and RT, were identified as significant factors in the CCD experimental design, exerting a meaningful influence on both the DH and antioxidant capacity (*p* < 0.05). The lack-of-fit test further confirmed the validity of the models, with Prob > F values of 3.84 for DH and 1.21 for antioxidant capacity, both indicating a non-significant lack of fit (*p* > 0.05) relative to the pure error. These results demonstrate that the proposed linear models adequately describe the experimental data and can be reliably used to predict process responses under the tested conditions.

Overall, the linear response surface approach effectively captured the relationship between the process variables and the responses, demonstrating its suitability for predicting and optimizing enzymatic hydrolysis efficiency and associated *in vitro* antioxidant activity in *U. rigida* protein hydrolysates.

Analysis of the response surface plots enabled prediction of the effects of the E/S ratio and RT on both DH and antioxidant activity. DH is often associated with the generation of bioactive peptides; therefore, in the present study, DH was used strictly as an indicator of hydrolysis progression rather than as a direct biological response. Previous studies have nonetheless reported strong associations between the extent of hydrolysis and antioxidant potential of protein-derived peptides (Baek and Cadwallader, 1995).

For antioxidant activity, a clear positive trend was observed: both increasing E/S ratio and RT resulted in higher ORAC values (Figure 2). The optimal conditions predicted by the model yielded an antioxidant capacity of 2.41 ± 0.06 μmol_Trolox equivalents_/mg_hydrolysed protein_, and these conditions were subsequently validated experimentally. Considering that antioxidant peptides are typically smaller than 3 kDa (Khasmakhi et al., 2025; Tavares et al., 2011), the hydrolysates obtained under optimal conditions were fractionated by ultrafiltration using a hydrophilic 3 kDa membrane. Antioxidant activity was then quantified in the total hydrolysate, the < 3 kDa permeate, and the > 3 kDa retentate. The experimentally determined activity of the total hydrolysate (2.6 ± 0.4 μmol_Trolox equivalents_/mg_hydrolysed protein_) fell within the 95% confidence interval of the model predictions, confirming the adequacy of the fitted model.

As expected, the < 3 kDa fraction displayed the highest antioxidant activity (4.8 ± 0.3 μmol_Trolox equivalents_/mg_hydrolysed protein_), markedly exceeding both the total hydrolysate and the > 3 kDa fraction (1.3 ± 0.1 μmol_Trolox equivalents_/mg_hydrolysed protein_). This result reinforces the well-established observation that low-molecular-weight peptides are primarily responsible for antioxidant activity in marine and food protein hydrolysates.

The antioxidant activities obtained here compare favorably with values reported for other macroalgal protein hydrolysates, such as *Laminaria ochroleuca* (2.5 μmol_Trolox equivalents_/mg_hydrolysed protein_) (Andrade et al., 2021) and *Porphyra dioica* (0.96 μmol_Trolox equivalents_/mg_hydrolysed protein_) (Minekus et al., 2014), and are within the range typically described for food-derived hydrolysates, including whey proteins and ovotransferrin (0.7–3.0 μmol_Trolox equivalents_/mg_hydrolysed protein_) (Contreras et al., 2011; Huang et al., 2010; Santana et al., 2016). These findings indicate that the *U. rigida* peptide fractions are capable of scavenging peroxyl radicals under controlled experimental conditions. Such performance could reflects the presence of peptides enriched in branched-chain amino acids (valine, leucine, isoleucine) and aromatic residues (tyrosine, tryptophan, phenylalanine), whose indole and phenolic groups efficiently donate hydrogen atoms (Ajibola et al., 2011; Baek et al., 2024; Xu et al., 2017), thereby enhancing radical-scavenging capacity. However, it is important to note that the ORAC assay reflects an *in vitro* estimate of antioxidant potential and does not necessarily translate into biological efficacy *in vivo*. Accordingly, while the results support the potential bioactivity of these low-molecular-weight peptides, further investigation in cellular systems and animal models will be needed to clarify their physiological relevance.

### 3.3 Anti-amyloidogenic activity of the peptide extract concentrate

**FIGURE 3:**
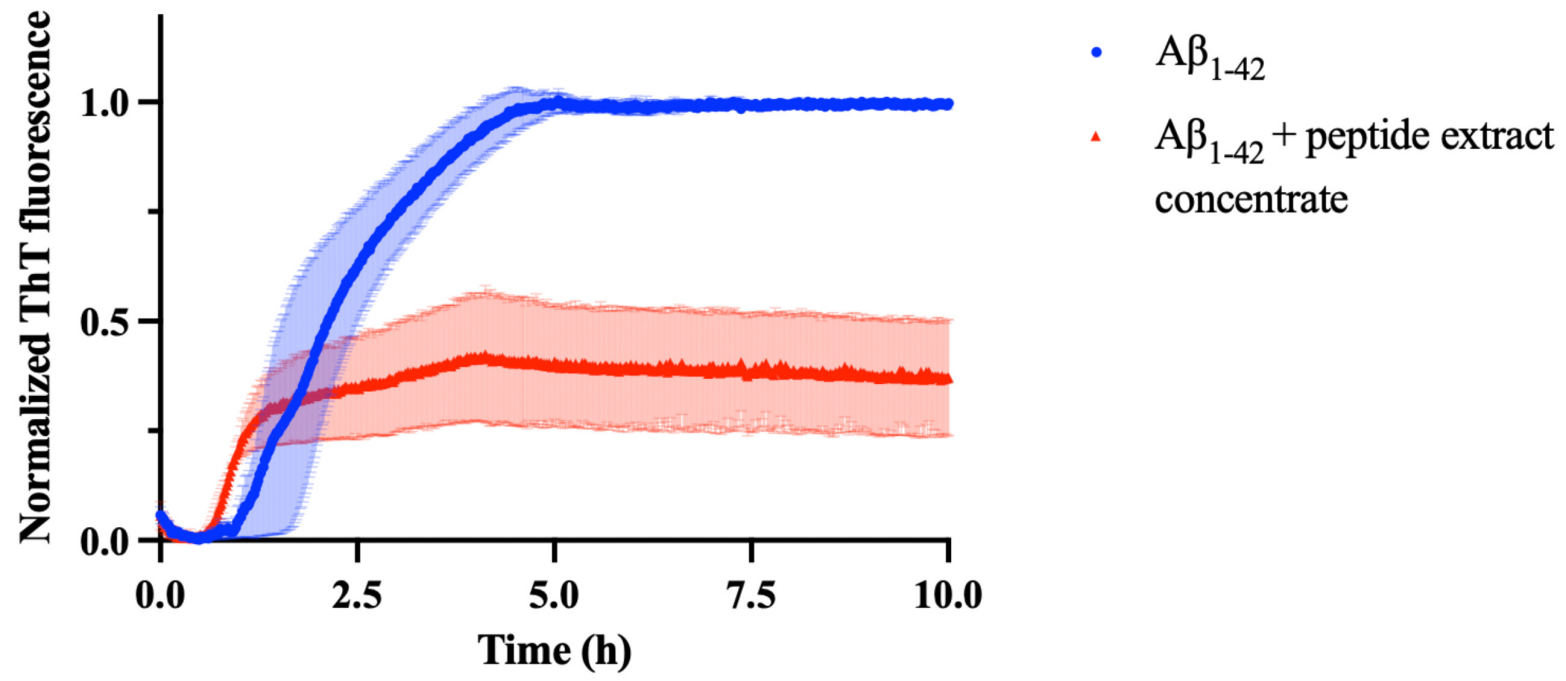
Normalized ThT fluorescence of amyloid beta peptide (Aβ)_1–42_ (25 μM) as a function of time (h) after incubation of Aβ monomers in the absence (blue) and in the presence of the *U. rigida* peptide concentrate extract (red) at a ratio of 1:25 (Aβ_1–42_ to peptide extract concentrate).

This study was specifically designed as a targeted and mechanistic proof-of-concept focusing on Aβ aggregation. While Aβ aggregation represents a central and well-established pathological hallmark of AD, disease progression also involves additional processes, including tau pathology, neuroinflammation, and synaptic dysfunction, which were beyond the scope of the present work. The inhibitory *in vitro* potential of peptide extract concentrate (molecular weight < 3 kDa) on the aggregation of Aβ_1–42_ was investigated to evaluate its possible role in delaying or preventing the progression of AD. The experiment aimed to determine whether the peptide extract concentrate could interfere with the fibrillogenesis process of Aβ_1–42_. Based on previous reports describing the use of similar inhibitor-to-Aβ ratios for effective inhibition of amyloid aggregation (Horsley et al., 2020), an Aβ-to-peptide extract concentrate ratio of 1:25 was selected to ensure sufficient peptide concentration for measurable interference with fibril formation. The kinetics of Aβ fibril formation were monitored using ThT fluorescence spectroscopy, a widely used method for detecting β-sheet–rich amyloid structures (Andrade et al., 2022a). This exhibit enhanced fluorescence upon binding to β-sheet conformations, which are characteristic of mature amyloid fibrils. Therefore, the fluorescence intensity directly correlates with the quantity of fibrillar aggregates present in the sample (Xue et al., 2017). In this assay, the Aβ_1–42_ peptide was incubated under aggregation-promoting conditions in the presence and absence of the *U. rigida* peptide extract, and ThT fluorescence was recorded over time to evaluate the effect of the extract on fibril formation kinetics. Figure 3 displays the normalized ThT fluorescence curves.

It can be confirmed from Figure 3 that the curves exhibit a standard sigmoidal pattern, which is typical of the amyloid peptides aggregation kinetics and was previously reported in other works (Andrade et al., 2021). This sigmoidal behavior reflects the multi-phase process of fibril formation, encompassing the lag, elongation, and stationary phases. At the onset of the aggregation process, ThT fluorescence is minimal, indicating that the samples contain very few β-sheet structures. This initial lag phase corresponds to the nucleation stage, during which monomeric Aβ_1–42_ peptides interact and assemble into small oligomeric species that serve as nuclei for subsequent fibril growth. Following the lag phase, the fluorescence intensity rises sharply, marking the elongation phase. This phase reflects the rapid conversion of oligomers into mature β-sheet–rich fibrils, with a concomitant increase in ThT binding. Eventually, the aggregation process reaches a plateau, or stationary phase, when most of the peptide has been organized into fibrillar aggregates and fibril growth approaches equilibrium.

Through the application of Equations 2, 3, the kinetic parameters corresponding to each ThT curve were determined and are listed in Table 3.

**TABLE 3:** Kinetic parameters derived from normalized ThT fluorescence curves for Aβ aggregation in the absence and in the presence of *U. rigida* peptide concentrate extract.

Kinetic parameters	Aβ	Aβ + Peptide concentrate extract
Max. Fluorescence	0.988 ± 0.005	0.32 ± 0.5 *
t_lag_ (h)	1.7 ± 0.3	0.72 ± 0.3*
k (h^–1^)	1.91 ± 0.04	4.5 ± 0.7*
t_1/2_ (h)	2.2 ± 0.3	0.95 ± 0.07*

*p < 0.05 suggests a statistical difference compared to the control.

Notably, in the presence of the *U. rigida* protein extract, several alterations in the aggregation kinetics were observed. The lag phase was significantly shorter (0.72 ± 0.30 h) than that of Aβ alone (1.7 ± 0.3 h), suggesting that the extract modulates the earliest stages of peptide association. The extract also increased the apparent elongation rate constant from 1.91 ± 0.04 h^−1^ to 4.5 ± 0.7 h^−1^, resulting in a markedly reduced half-time (0.95 ± 0.07 h vs. 2.2 ± 0.3 h for the control). In addition, the overall ThT fluorescence intensity was substantially lower in the treated samples (0.32 ± 0.05) compared to Aβ alone (0.988 ± 0.005), indicating a reduced accumulation of β-sheet–rich fibrillar structures. This finding suggests that peptide extract concentrate in the extract reduces ThT signal under the tested conditions and alters Aβ aggregation kinetics Aβ fibrillogenesis *in vitro*, potentially by interacting with monomers or oligomers to prevent their self-assembly, ultimately leading to a reduced formation of Aβ fibrils.

These observations are consistent with other studies demonstrating the anti-amyloidogenic effects of several synthetic peptides. Among them, the pentapeptide KLVFF (Aβ_16–20_), corresponding to the central hydrophobic core of Aβ, has been shown to specifically bind to the homologous region of the native peptide, thereby blocking nucleation and fibril elongation (Tjernberg et al., 1996). A modified analog, LPFFD (iAβ_5_), functions as a β-sheet breaker peptide, disrupting β-sheet conformations and promoting both inhibition of fibrillogenesis and disaggregation of preformed fibrils, leading to reduced amyloid burden in murine models (Soto et al., 1998).

Additionally, some other compounds present in the seaweed, such as polyphenols, exhibit antiamyloidogenic properties. For example, a study conducted by Andrade et al., found that the natural compound caffeic acid also significantly decreases the amount of Aβ_1–42_ fibrils (Andrade et al., 2023). Similarly, polyphenols such as resveratrol and epigallocatechin-3-gallate have been shown to inhibit Aβ fibrillogenesis by stabilizing non-toxic oligomers and preventing β-sheet formation (Martins et al., 2020; Sharma et al., 2023). Collectively, these findings reinforce the potential of naturally derived bioactive molecules as promising candidates for therapeutic strategies targeting amyloid-related pathologies in AD.

### 3.4 Nanoparticles loaded with peptide extract concentrate

**FIGURE 4:**
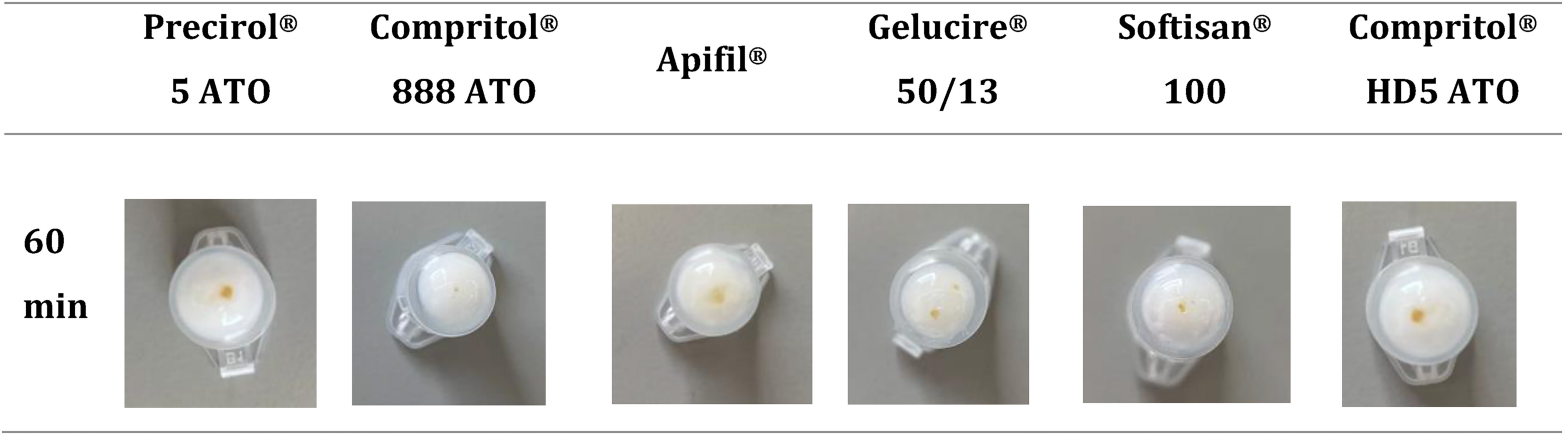
Appearance of the mixture of solid lipids and peptide concentrate extract from *U. rigida* at the mass ratio of 50:1 (w/w) after 1 h at 80 °C.

A lipid screening study was performed to evaluate the compatibility between the *U. rigida* peptide concentrated extract and different solid lipids. The main goal was to identify which lipid exhibited the highest solubilization capacity for the peptide-concentrated extract, ensuring its suitability for the development of SLNs. For this purpose, each lipid was mixed with the extract and maintained at 80 °C for 1 h. After this period, the visual appearance of the mixtures was evaluated to determine the extent of peptide-concentrated extract dissolution (Figure 4).

Among the lipids tested, Compritol^®^ 888 ATO showed the highest solubilization capacity for the peptide concentrate extract, resulting in a more homogeneous mixture after 1 h of incubation. Softisan^®^ 100 also demonstrated partial solubilization; however, undissolved residues of the extract were still visible, indicating incomplete compatibility. The remaining tested lipids exhibited poor or negligible solubilization of the extract under the same conditions.

Based on these observations, Compritol^®^ 888 ATO was selected for the subsequent preparation of SLNs, due to its superior capacity to dissolve the extract and its well-known biocompatibility and stability properties (Ilyas et al., 2022). Following this selection, SLNs were produced to encapsulate the peptide concentrate using Pluronic F-127 as an emulsifier, and their physicochemical characteristics were thoroughly evaluated. The NPs were assessed in terms of mean particle size, PdI, and zeta potential, providing insights into their uniformity, colloidal stability, and surface charge. The results for both unloaded and extract-loaded SLNs are summarized in Table 4.

**TABLE 4:** Physicochemical features of unloaded solid lipid nanoparticles (SLNs) and peptide concentrate extract-loaded SLN. Data is presented as mean ± SD (*n* = 3).

	Size (nm)	PdI	Zeta potential (mV)	EE (%)	LC (%)
Unloaded SLNs	116 ± 1	0.16 ± 0.02	−18.4 ± 1.9	–	–
Peptide concentrate extract-loaded SLNs	158 ± 14 *	0.23 ± 0.07	−4.7 ± 0.6 *	54.1 ± 0.1	10.4 ± 0.1

*p < 0.05 suggests a statistical difference compared to the control unloaded SLNs.

Table 4 demonstrates that the selected lipid and formulation method produced SLNs with consistent size, low PDI, and appropriate zeta potential, indicating their suitability for biomedical applications. NPs to be used as drug delivery systems should have a mean size lower than 200 nm, a PdI of less than or equal to 0.2, a negative/neutral zeta potential, and good colloidal stability (Xu et al., 2021). In this case, peptide concentrate extract-loaded NPs have average diameters of 158 ± 14 nm, which makes them suitable for administration to humans. As reported by other authors, nanoparticles larger than 200 nm tend to be rapidly eliminated by the phagocytic system, whereas those below this size are more suitable biotive compounds delivery (Lee et al., 2022). An increase in the SLN diameter of around 42 nm was observed after the extract encapsulation (*p* < 0.05), indicating a rearrangement between the solid lipid and the peptide concentrate extract. In a previous study developed by Loureiro et al., an increase in the SLN diameter of around 35 nm was observed also when the NPs were encapsulated in grape skin or grape seed extracts (Loureiro et al., 2017).

The PdI is a measure used in particle size distribution analysis to define the range of particle sizes. “Polydispersity” refers to the degree of non-uniformity in a particle size distribution (Xu et al., 2021). The unloaded nanoparticles present a PDI of 0.16 ± 0.02, indicating a homogeneous population. In the case of the peptide concentrate extract-loaded SLNs, no statistically significant differences were observed. The PDI is 0.23 ± 0.07, indicating that the nanoformulation is homogeneous and do not contain aggregates when loaded with the bioactive mixture (Loureiro and Pereira, 2021).

Finally, zeta potentials > 30 mV or <−30 mV are considered adequate to ensure electrostatic stability. However, for the purpose of distributing the drug in the human body, the particles should have an almost neutral charge, i.e., for the zeta potential to be as close to 0 as possible or slightly negative to avoid cytotoxicity (Xu et al., 2021). It is well known that cationic particles, which are positively charged, interact more effectively with cell membranes compared to anionic and neutral particles, thereby enhancing intracellular uptake (Andrade et al., 2022b). However, this same characteristic also increases the toxicity associated with cationic particles, as they are more likely to cause cellular damage and/or disrupt lysosomal membranes (Augustine et al., 2020). Here, the NPs loaded with the peptide concentrate have a zeta potential of −4.7 ± 0.6 mV, therefore expected to be safe in terms of cytotoxicity, which is favorable for distributing the extract in the human body. This value is significantly lower than the unloaded nanoparticles (−18.4 ± 1.9 mV), which can mean that can means than some of the peptide extract concentrate could be localized in the nanoparticles’ surface.

The amount of peptide concentrate extract encapsulated was 54.1 ± 0.1 %. From this result, we conclude that SLNs are able to moderately encapsulate the peptide concentrate extract from *U. rigida*. Similarly, in a previous study, SLNs were loaded with a peptide, and the obtained EE was 50.4 ± 3.0 % (Hu et al., 2003), which is close to the value obtained for the SLNs under study. The LC of the NPs was also calculated, and the result obtained was 10.4 ± 0.1%, aligning with those reported in the literature, which indicate that SLN nanoparticles generally exhibit low loading capacities (Mehta et al., 2023). Solid and crystalline structures tend to accommodate less space for the active molecule compared to more imperfect systems or those with liquid lipids.

**FIGURE 5:**
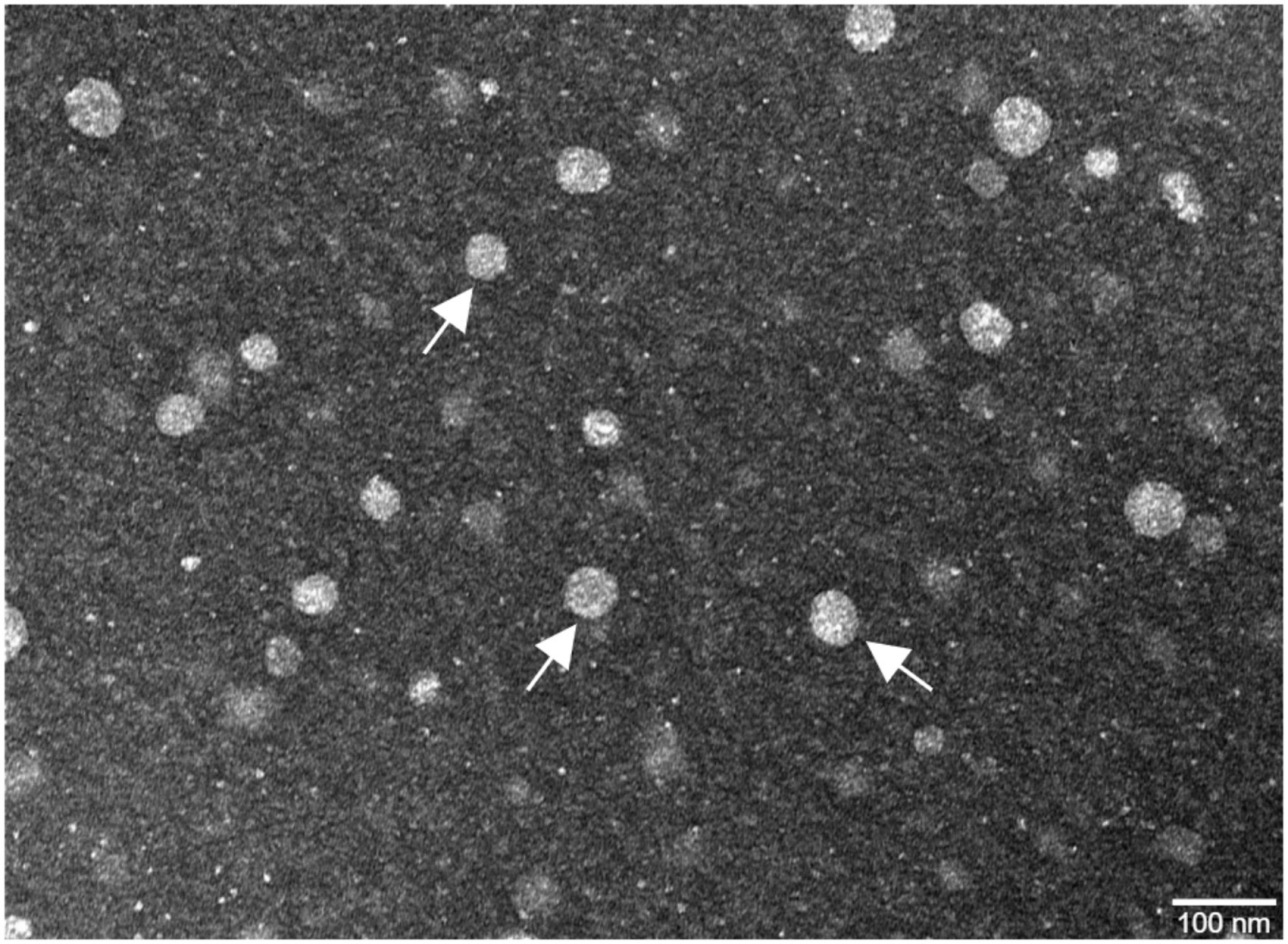
Transmission electron microscopy (TEM) images of SLNs with the encapsulated peptide concentrate extract. The scale bar corresponds to 100 nm. The arrows indicate some of the SLNs.

Additionally, the morphology of the peptide concentrate extract-loaded SLN was examined by TEM, allowing visualization of particle shape, surface smoothness, and the presence of any aggregation. Figure 5 shows the images obtained, in which arrows have been placed to indicate some of the NPs.

The TEM image revealed that the SLNs exhibited the expected spherical morphology. The observed particle sizes are notably smaller than the values obtained by DLS analysis. This discrepancy is attributable to the sample preparation method for TEM, which undergoes a dehydration process that can affect particle size. In contrast with DLS measures the hydrodynamic diameter of NPs, which is influenced by factors such as the presence of hydration layers surrounding the NP (Souza et al., 2016; Wilson and Prud’homme, 2021). As a result, DLS measurements often overestimate the actual particle size compared to TEM observations. Also, the fact that some are larger than others can be explained by the PdI associated with each sample, since polydispersity leads to different particle sizes within the same sample.

### 3.5 Solid lipid nanoparticles stability

#### 3.5.1 Stability under simulated gastrointestinal environment

Oral delivery was selected as the primary administration route because it enables chronic intake, which is essential for interventions targeting slow-progressing conditions such as AD. Although oral peptides typically exhibit limited bioavailability, nanoencapsulation in SLNs can partially overcome gastrointestinal degradation, enhance mucosal permeation, and increase lymphatic uptake, thereby increasing the fraction of peptides that reaches systemic circulation (Mehrdadi, 2024). The NP’s stability was assessed in the gastric and intestinal simulated buffer to simulate the real conditions that the NPs will encounter in the human body after oral administration, providing a more accurate prediction of the NP’s behavior in clinical use. For that, different media simulating the pHs and enzyme content of the stomach and intestine were used. So, testing the NPs in these media helps to understand whether they are resistant to different pHs and enzymatic degradation.

The NPs were in contact with the FaSSGF buffer (pH 1.2) simulating the gastric environment for 2 h, and after that, they were in contact with the FaSSIF buffer (pH 6.5) simulating the intestinal medium for 3 h. To understand whether they were stable in these media, their size, PdI, and zeta potential were measured before and after being subjected to each of the media. The results obtained are shown in Table 5.

**TABLE 5:** Physicochemical features of peptide concentrate extract-loaded SLNs before and after passing through FaSSGF buffer simulating the gastric environment for 2 h, and FaSSIF buffer simulating the intestinal medium for 3 h. Data is presented as mean ± SD (*n* = 3).

	Size (nm)	PdI	Zeta potential (mV)
Control in H_2_O	150 ± 13	0.27 ± 0.01	−4.1 ± 0.6
After 2 h in FaSSGF	146 ± 11	0.24 ± 0.03	−0.9 ± 0.2 *
After 3 h in FaSSIF	146 ± 13	0.26 ± 0.03	−1.3 ± 0.2 *

*p < 0.05 suggests a statistical difference compared to the control in ultrapure water.

Looking at the results in Table 5, it can be seen that the SLNs loaded with the peptide concentrate extract remain stable after being subjected to each of the media, with no significant variation in the average diameter of the NPs (*p* > 0.05) or the PdI (*p* > 0.05). The zeta potential exhibited a significant variation in values (*p* < 0.05), however, under simulated gastric and intestinal conditions, the zeta potential approached neutrality. This reduction in zeta potential values is attributable to the acidic pH of these gastrointestinal simulated media. When dispersed in FaSSGF, the zeta potential shifted from −4.1 ± 0.6 to −0.9 ± 0.2 mV. This reduction is consistent with the known effect of acidic gastrointestinal conditions on nanoparticle surface charge. The low pH promotes protonation of negatively charged groups, while the presence of electrolytes compresses the electrical double layer and contributes to partial neutralization of surface charges, thus decreasing the magnitude of zeta potential (Yokel et al., 2019). This effect looks reversible because after being in contact with FaSSIF media, it presents a tendency to increase again from −0.9 ± 0.2 to −1.3 ± 0.2 mV. Those variations were also observed in a study where polymeric NPs were used to transport vitamin B9 and vitamin B12 orally (Ramalho et al., 2021). Therefore, it can be concluded that the particles are stable when exposed to the gastric and intestinal simulated environments and do not undergo physical degradation.

#### 3.5.2 Stability over time under storage conditions

**FIGURE 6:**
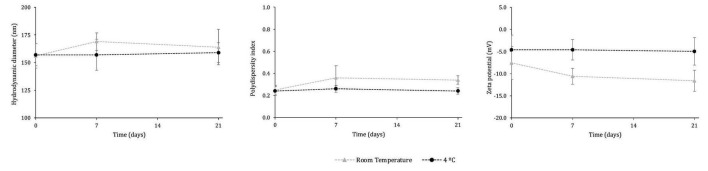
Physicochemical features of the peptide concentrate extract-loaded SLNs over 21 days at room temperature and 4 °C. No significant differences (*p* > 0.05) were observed over time compared with time 0 h.

The long-term stability of SLNs is a key criterion for their potential future utilization, as it ensures that the physicochemical properties of the nanoformulation are preserved throughout storage. To assess this, the peptide extract-loaded SLNs were stored for 3 weeks under two conditions, 4 °C and room temperature, and periodically analyzed for their hydrodynamic diameter, PdI, and zeta potential (Figure 6).

Statistical analysis showed no significant differences (*p* > 0.05) in any of the measured parameters over 21 days when the formulations were stored at 4 °C, indicating that their physicochemical characteristics remained unchanged during this period. The samples also retained a homogeneous visual appearance with no evidence of phase separation, flocculation, or creaming, further supporting the stability of the formulations. Similarly, at room temperature, no significant variations (*p* > 0.05) were observed after 3 weeks of storage, demonstrating that the SLNs maintained their size distribution, surface charge, and general integrity. Overall, these results confirm that the SLNs, whether containing the peptide extract or not, exhibit good storage stability under the tested conditions.

## 4 Conclusion

In this work, an experimental design approach was successfully employed to optimize the extraction and hydrolysis conditions of *U. rigida* peptides with *in vitro* antioxidant and anti-amyloidogenic potential. Peptide fractions were primarily classified by molecular weight (<3 kDa), a common initial step to enrich for potentially bioactive peptides. However, in the future, further characterization using techniques such as LC–MS/MS will be necessary to identify the specific peptides responsible for the observed effects. The optimized extract, particularly the peptide fraction below 3 kDa, demonstrated strong radical-scavenging activity and a remarkable ability to inhibit the Aβ_1–42_ aggregation, indicating its potential relevance as a neuroprotective candidate. Both activities were assessed because AD is multifactorial, and there is growing interest in multifunctional bioactive peptides. To address the inherent limitations of peptide stability and bioavailability, the peptide concentrate extract was encapsulated in SLNs. The SLNs were successfully developed using Compritol^®^ 888 ATO and Pluronic F-127 to encapsulate the < 3 kDa peptide fraction. The formulated SLNs demonstrated spherical morphology, appropriate size, low polydispersity, stable zeta potential, and good encapsulation efficiency, as well as stability under both storage and simulated gastrointestinal conditions, confirming their suitability as delivery vehicles for oral administration.

Future studies should focus on peptide characterization, *in vivo* evaluation, further nanoformulation optimization, and elucidation of the molecular mechanisms underlying the observed antioxidant and anti-amyloidogenic effects, as well as their functional links. Collectively, this approach provides a promising platform for encapsulating peptide extract concentrates, supporting their continued preclinical evaluation in the context of amyloid-related diseases.

## Data Availability

The raw data supporting the conclusions of this article will be made available by the authors upon request.
